# SLAT/Def6 plays a critical role in the pathogenic process of experimental autoimmune uveitis (EAU)

**Published:** 2012-07-07

**Authors:** Barbara P. Vistica, Guangpu Shi, Lindsey Nugent, Cuiyan Tan, Amnon Altman, Igal Gery

**Affiliations:** 1 Laboratory of Immunology, National Eye Institute, NIH, Bethesda, MD; 2 La Jolla Institute for Allergy and Immunology, La Jolla, CA

## Abstract

**Purpose:**

SWAP 70-like adaptor of T cells (SLAT; aka Def6) is a recently discovered guanine nucleotide exchange factor for Rho guanosine triphosphate (GTP)ases that has been previously shown to play a role in cluster of differentiation(CD)4+ T cell activation, T-helper (Th)1/Th2/Th17 differentiation and development of experimental autoimmune encephalomyelitis. Here, we investigated the role of SLAT/Def6 in the development of experimental autoimmune uveitis (EAU), an animal model for several uveitic conditions in humans.

**Methods:**

SLAT/Def6 deficient (“KO”) mice and C57BL/6 controls were immunized with interphotoreceptor retinoid-binding protein (IRBP), along with pertussis toxin. The development of ocular inflammation was determined by both fundoscopy and histological examination. Lymphoid cells from draining lymph nodes were cultured with IRBP to measure lymphocyte proliferation and release of cytokines. Purified dendritic cells were tested for their capacity to present antigen to responding lymphocytes. In addition, the lymphoid cells were tested for the expression of forkhead box P3 (FoxP3), using conventional methods, and the activity of T-regulatory cells was determined by their capacity to inhibit in vitro proliferative responses. Serum anti -IRBP antibody levels were measured by enzyme-linked immonosorbant assay (ELISA). quantitative polymerase chain reaction (qPCR) was used to determine the transcript levels of cytokines in inflamed eyes.

**Results:**

SLAT/Def6 KO mice had significantly reduced EAU compared to controls. Cells isolated from draining lymph nodes of SLAT/Def6 KO mice exhibited impaired proliferation and production of Th1 and Th17 signature cytokines (interferon [IFN]-γ and interleukin [IL]-17, respectively) when compared with cells isolated from control mice. qPCR of inflamed eyes detected similar levels of *IFN-γ* transcript in control and SLAT/Def6 KO mice, whereas the *IL-17* transcript levels in eyes of the SLAT/Def6 KO mice were lower than in eyes of the controls. The SLAT/Def6 KO mice resembled their wild type (WT) controls, however, in the levels of their serum antibody against IRBP, the antigen presenting capacity of their dendritic cells, the proportion of cells expressing Foxp3 and the immunosuppressive activity of their T-regulatory cells.

**Conclusions:**

SLAT/Def6 KO mice exhibit reduced capacity to develop ocular inflammation and cellular activity when immunized with IRBP. Our study provides new data showing that SLAT/Def6 plays a major role in the T cell-mediated autoimmune processes that bring about the inflammatory eye disease, EAU.

## Introduction

SWAP 70-like adaptor of T cells (SLAT), also named Differentially expressed in FDCP-6 homolog (Def6), has strong homology with switch-associated protein 70 (SWAP-70), a B-cell protein involved in B-cell activation, Ig class switching and migration to lymphoid organs [1]. SLAT/Def6 is a protein that regulates many T cell processes such as cluster of differentiation (CD)4+ activation and T-helper (Th)1/Th2/Th17 differentiation in vitro and in vivo [2-4]. SLAT/Def6 is abundant in central and peripheral lymphoid tissues, with high amounts found in thymocytes and peripheral T cells. Recently, SLAT/Def6 has been shown to play a major role in the development and pathogenesis of Th17 cell-mediated experimental autoimmune encephalomyelitis (EAE) [3]. However, an earlier study described enhanced rheumatoid arthritis-like joint disease in Def6 deficient mice [5], although questions were later raised about the mixed background of the mice affecting the results [6]. Here, we investigated the role of SLAT/Def6 in the development of experimental autoimmune uveitis (EAU), an animal model for several uveitic conditions in humans [7-9]. EAU is a T cell-mediated disease induced in mice by immunization with the retinal antigen, interphotoreceptor retinoid-binding protein (IRBP) [7-9]. To examine the involvement of SLAT/Def6 in the pathogenic process of EAU, we compared SLAT/Def6 deficient mice with wild-type (WT) controls for their susceptibility to EAU induction and for their capacity to develop an immune response against IRBP. The SLAT/Def6 deficient animals exhibited lower susceptibility to the disease and reduction in their proliferation and pro-inflammatory cytokine profile in response to IRBP. Our data, thus, supports the notion that SLAT/Def6 may be a promising drug target for T cell-mediated autoimmunity and inflammation.

## Methods

### Mice

SLAT/Def6 deficient (“KO”) mice on a C57BL/6 background have been previously described [4]. C57BL/6J mice were purchased from The Jackson Laboratory. SLAT/Def6 KO mice and age-and gender-matched control C57BL/6 mice, between the ages of 8 and 12 weeks, were used in this study. The mice were housed in a pathogen-free facility and all experiments were performed under protocols approved by the Animal Care and Use Committee of the National Eye Institute, NIH.

### Induction and evaluation of EAU

SLAT/Def6 KO mice and their C57BL/6 controls were immunized with bovine IRBP (150 μg), emulsified in complete Freund’s adjuvant (CFA), administered subcutaneously [10]. In addition, the mice were injected intraperitoneally with 0.2 μg pertussis toxin (List Biological Laboratories, Inc., Campbell, CA). The development of ocular inflammation was determined by fundoscopy on day 12 post-immunization and by histological examination on day 14, following euthanization. Severity of disease, on a scale of 0–4, in half point increments, was scored as detailed elsewhere [11,12].

### Lymphocyte responses: Proliferation assay

Lymphoid cells from draining lymph nodes (dLN) were collected 14 days post-immunization and pooled within each group. LN cells were cultured as detailed elsewhere [13]. Briefly, draining lymph node cells were collected 14 days post-immunization and cultured in triplicate in flat-bottomed 96-well plates, at 4×10^5^ cells in RPMI-1640 medium, supplemented with HL-1 (BioWhittaker, Walkersville, MD), 2-mercaptoethanol (50 µM), and antibiotics. The stimulants included IRBP at several concentrations and purified protein derivative (PPD), a component of CFA. Following incubation for 72 h, the cultures were pulsed with [3H]-thymidine (0·5 µCi/10 µl/well) for an additional 16 h. Data are presented as mean delta counts per minute (Δcpm)= cpm in stimulated cultures minus cpm in unstimulated cultures.

### Dendritic cell (DC) functional assay

DC were isolated from spleens of WT and KO mice by digestion with collagenase D and magnetic separation with autoMACS (Miltenyi Biotec, Auburn, CA) [14]. IRBP responder cells (CD4+) were isolated from spleens and LN of IRBP-immunized mice (day 7) and cultured with the WT or KO DC for antigen presentation, alone, or with stimuli, phytohemagglutinin (PHA), or different concentrations of IRBP. Proliferation of the cultures was measured as detailed in [13].

### Lymphocyte responses: Cytokine production

dLN cells were cultured in 24-well plates at 5×10^6^ cells/well in 1 ml of RPMI 1640 medium, supplemented with 2% HL-1 serum replacement (Lonza, Walkesville, MD), antibiotics, and 2-mercaptoethanol. The cultures were stimulated with whole IRBP at 10 μg/ml. Supernatants were collected following incubation for 48 h and their levels of interferon (IFN)-γ and interleukin (IL)-17 were determined by enzyme-linked immunosorbent assay (ELISA) kits (R&D Systems, Minneapolis, MN).

### Expression of FoxP3 by lymph node cells

Lymph node cells of the dLN were also tested for their expression of the transcription factor specific for T-regulatory (Treg) cells, FoxP3, using the method described in [15,16]. Briefly, isolated dLN cells were fixed and permeabilized with the Fixation/Permeabilization buffer for 1 h at 4 °C before intracellular staining with allophycocyanin-conjugated anti-Foxp3 antibody, following the procedure recommended by the manufacturer (eBioscience, San Diego, CA).

### Serum antibody levels

Mouse sera collected on day 8 and 14 post-immunization were tested for the level of antibody to IRBP by ELISA [17].

### qPCR Analysis

qPCR was used to compare the transcript levels of immune-related molecules in inflamed eyes, using the procedure detailed elsewhere [18]. Data obtained were normalized with values of β-actin and calculated to obtain relative expression values.

### T-regulatory (Treg) cell functional assay

Tregs (CD4+ CD25+) were sorted by FACS from spleens and LN of WT and KO mice. Naïve responder cells (CD4+, CD25-) were FACS sorted from naïve WT mice and activated with soluble anti-CD3 antibody and APCs (CD3-depleted, irradiated). Tregs from WT or KO mice were cultured alone, with the stimuli and APC, or added in various ratios to cultures of naïve responder cells to test for the ability of the Tregs to block proliferation of the responder cells.

### Statistics

Unpaired, two-tailed *t* test was performed for comparison of severity of disease, proliferative responses, and cytokine analyses. ns=not significant; *p<0.05; **p<0.005.

## Results

### Mice deficient in SLAT/Def6 are poor responders to EAU induction

**Figure 1:**
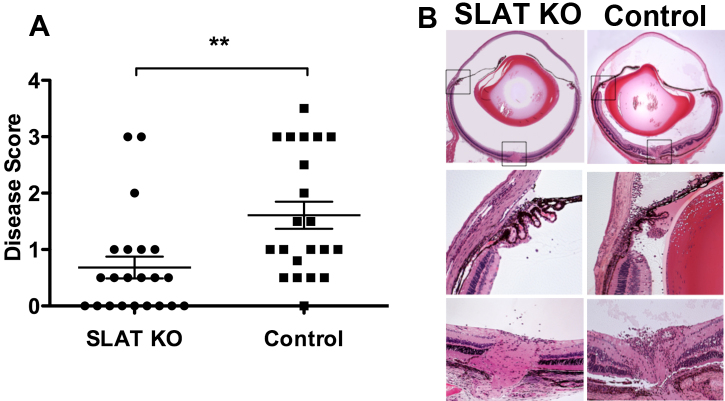
SLAT/Def6 deficient mice are inferior to their WT controls in developing EAU. **A**: Disease scores of individual mice of the two mouse lines. Horizontal lines are mean ±SEM. A summary of repeated experiments comparing the response in mice of the two lines. ** p<0.005. **B**: Sections of representative eyes of the two groups. The changes in the eye of the control mouse are markedly more severe and mainly include infiltration of inflammatory cells in various ocular tissues, as well as retinal folding and loss of photoreceptor cells (score: 2+). Only minor infiltration is seen in the SLAT/Def6 deficient mouse eye (score: 0.5).

To examine the susceptibility of SLAT/Def6 KO mice to induction of EAU, we immunized groups of these mice and their WT C57BL/6 controls with IRBP, as detailed in the Methods section. Development of EAU in the mouse eyes was determined by fundoscopy and histological examination, with good correlation between these two methods of disease detection. Data of histological analyses of repeated experiments are summarized in Figure 1A and show that the deficient mice were significantly inferior to their WT controls in developing EAU.

Figure 1B demonstrates histological sections of representative mouse eyes of the two groups. The WT control eye shows the typical EAU changes that include heavy infiltration of inflammatory cells throughout the optic nerve head, retinal vessels, and limbus, with numerous cells in the vitreous and anterior chamber. Retinal folding is also seen, as well as loss of photoreceptor cells. The eyes of the KO mouse, on the other hand, had very minimal inflammation compared to control eyes, with only a few inflammatory cells entering through the optic nerve head. Clinical changes in inflamed eyes, evaluated by fundoscopy, included swelling and inflammatory infiltrates at the optic nerve disc, cuffing and engorgement of retinal vessels, as well as retinal inflammatory lesions and scars.

### SLAT/Def6 KO mice are inferior in their lymphocyte responsiveness

**Figure 2:**
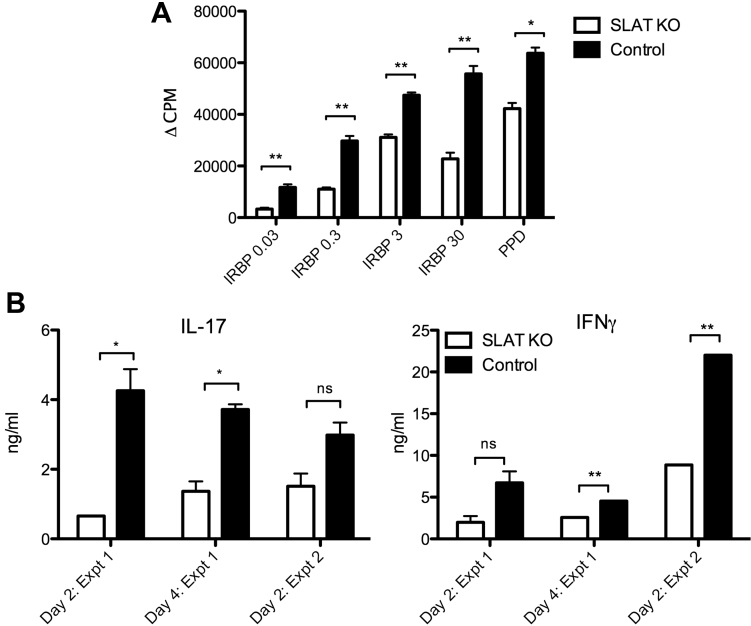
SLAT/Def6 KO mice are inferior to their WT controls in their cellular response to the immunizing antigens. **A**: Proliferative assay of a representative experiment; similar data were obtained in 2 other repeated experiments. IRBP was added at the indicated concentrations (μg/ml). PPD was added at 5 μg/ml. * p<0.05, **p<0.005. **B**: Levels of IL-17 and IFN-γ secreted by lymph node cells of SLAT/Def6 KO mice cultured with IRBP at 10 μg/ml. Data of two experiments, with supernatants collected on day 2 or 4 of culture. ns=not significant; *p<0.05.

Next, we compared the KO mice and their WT controls for their lymphocyte responsiveness toward IRBP, as well as PPD, a component of the CFA. Lymphoid cells collected from the dLN of mice of the two groups were collected from the mice euthanized on day 14 post-immunization and their responses were measured by the thymidine incorporation assay. Figure 2A is a representative experiment and shows that cells from the mice deficient in SLAT/Def6 responded with remarkably lower levels than their WT controls. Similar data were obtained in 2 other repeated experiments.

In addition, we determined in the lymphocyte culture supernatants the levels of IFN-γ and IL-17, the two signature cytokines for the Th1 and Th17 populations, respectively. As seen in Figure 2B, the levels of both cytokines were lower in cultures of the null mice as compared to cultures of the WT controls.

### Dendritic cells from SLAT/Def6 deficient mice resemble their WT controls in their antigen presenting capacity

**Figure 3:**
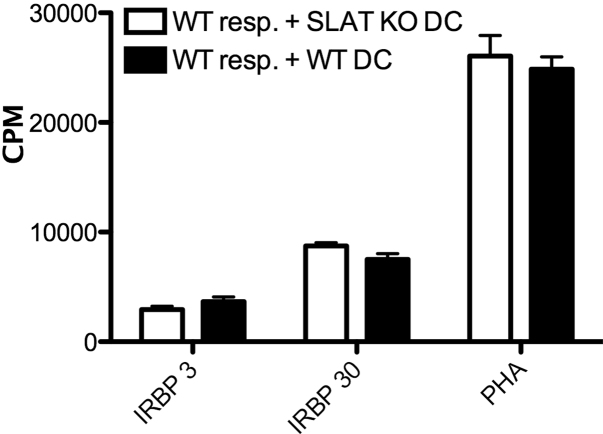
Unlike their defective lymphocyte responses, the SLAT/Def6 KO mice resemble their WT controls in their dendritic cell functional activity. DC preparations [14] from the KO mice or their WT controls were tested for their capacity to present IRBP to purified CD4 from syngeneic mice. The response was measured by thymidine incorporation assay [13].

We next compared the KO mice and their controls for another immunological parameter, i.e., the capacity to present antigen to T-cells. A representative experiment is shown in Figure 3. We used DC preparations from naïve mice of the two mouse lines [14] and added them to cultures of purified CD4 cells sensitized against IRBP, along with their target antigen. The CD4 cells responded to the antigen only when presented by the DC (not shown) and the DC from the KO mice resembled the DC from their WT controls in this capacity.

### Deficiency in SLAT/Def6 selectively affects the involvement of Th17 cells in the EAU pathogenic process

**Figure 4:**
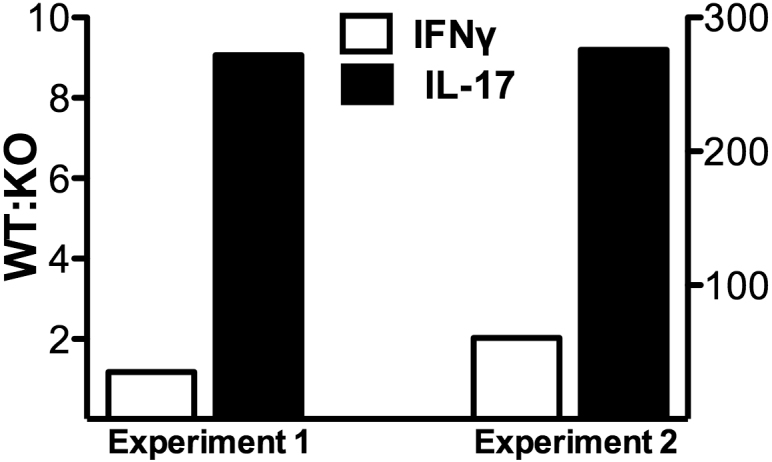
SLAT/Def6 deficiency selectively affects the involvement of Th17 cells in the EAU pathogenic process. Expression of IL-17 and IFN-γ was determined by qPCR in eyes of SLAT/Def6 KO mice and their WT controls. The data are expressed as the ratio between IL-17 and IFN-γ transcript levels in eyes of WT and KO mice, in each of the two experiments.

To further analyze the mode of action of the deficiency in SLAT/Def6, we compared eyes of KO and WT mice with EAU for the expression levels of IFN-γ and IL-17 transcripts. Data of two repeated experiments are shown in Figure 4. To overcome the variability in transcript levels determined by the qPCR method, we expressed the data as the ratios between the values of IL-17 and IFN-γ transcripts in eyes of WT and KO mice in each of the two experiments. As seen in Figure 4, the involvement of IL-17 in the pathogenic process of EAU was relatively lower in the SLAT/Def6 KO mice as compared with the controls.

### The poor cellular immune response in SLAT/Def6 KO mice is not due to increase in Treg activity

**Figure 5:**
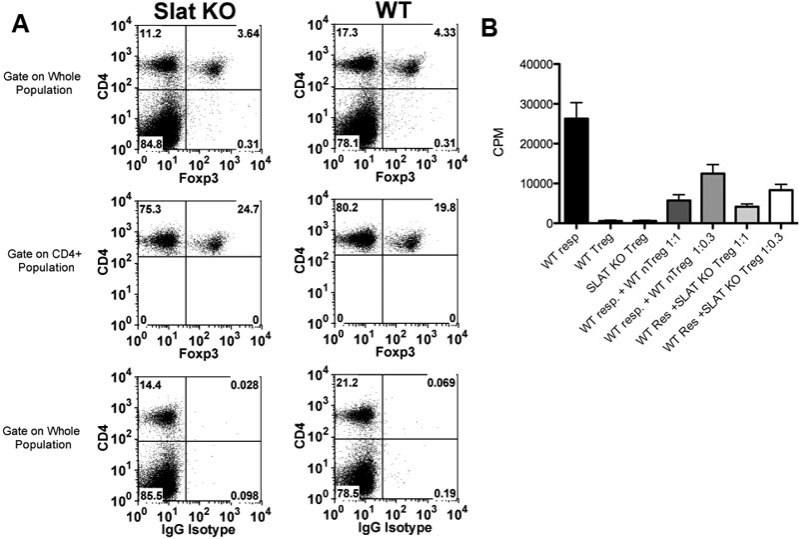
SLAT/Def6 KO mice resemble their WT controls in their Treg proportions and activities. **A**: Immunofluorescence analyses of lymph node cells in which the proportion of cells expressing FoxP3 was determined by gating on whole lymph node cells, or on the CD4 population. **B**: The Treg functional activity of the KO mice is similar to that of the WT controls.

Reduced immune response could be due to enhanced activity of Treg cells [18-20]. To examine this possibility in our system, we compared SLAT/Def6 KO mice and their WT controls for the proportions of T-cells that express FoxP3, a transcription factor specific to the majority of Treg cells [14]. The data of a representative experiment, shown in Figure 5A, indicate that the proportions of FoxP3 positive cells were similar among the draining lymph node populations from the immunized SLAT/Def6 KO mice and their controls. Likewise, the KO mice resembled their WT controls in the functional activity of their Treg cells (Figure 5B). Together, these data thus indicate that the possibility of enhanced Treg activity in the KO mice is unlikely.

### Deficiency in SLAT/Def6 does not affect the production of specific antibody

**Figure 6:**
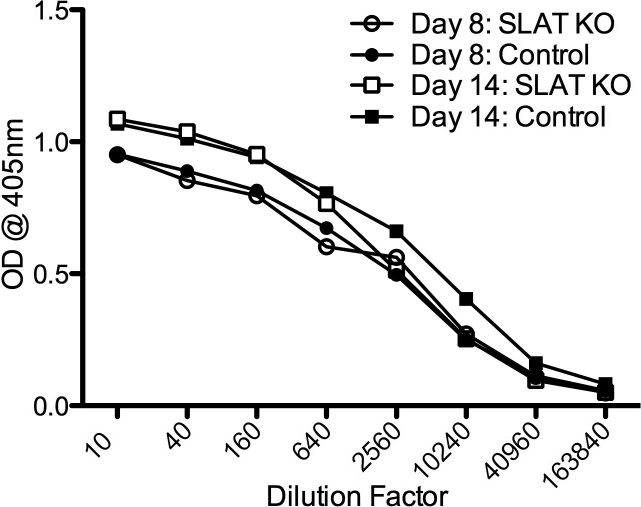
SLAT/Def6 KO mice resemble their WT controls in their production of anti-IRBP antibody. Sera were collected on days 8 or 14 post-immunization and the antibody levels were determined by ELISA.

Unlike the reduced specific cellular responsiveness in the SLAT/Def6 KO mice, negligible differences were noted between the deficient mice and their WT controls in their production of antibody against the immunizing antigen, IRBP. The data of repeated experiments are summarized in Figure 6 and show similar levels of antibody in sera collected from the two mouse groups on days 8 or 14 post-immunization.

## Discussion

When immunized with IRBP, SLAT/Def6 deficient mice exhibited remarkably reduced ocular inflammation and cellular immune responses as compared to their C57Bl/6 WT controls. A good correlation was seen between the clinical and histopathological changes that developed in eyes of mice of the deficient and WT control and the reduction in severity of these changes correlated well with lowered immune responses in the KO mice. Our data are in line with those of Canonigo-Balancio et al. [3], in which the SLAT/Def6 deficient mice showed resistance to development of EAE, another T-cell-mediated autoimmune disease. These two studies also provide data showing that the poor immune response in the SLAT/Def6 deficient mice cannot be attributed to increases in the proportion of Treg cells, or their functional activity. It is assumed, therefore, that the deficiency in SLAT/Def6 molecule affects the disease-inducing lymphocytes at one or more phases of their activation, migration to the target tissue, or capacity to initiate the pathogenic process. The finding that lymphocytes from the dLNs of the KO mice responded in culture less vigorously than their WT controls suggests a deficiency in the responsiveness to the specific antigen. In line with the study of Canonigo-Balancio et al. [3], responses in vitro of both Th1 and Th17 cells of SLAT/Def6 KO mice were lower than those of their WT controls (Figure 2).

Unlike the deficiency in their T-cell populations, SLAT/Def6 KO mice developed antibody against the immunizing antigen with levels similar to those of the WT controls (Figure 6). This finding indicates that the SLAT/Def6 molecules do not play a significant role in the process of antibody production. Likewise, the KO mice resembled their controls in the capacity of their dendritic cells to present antigen to T-cells.

In summary, SLAT/Def6 plays an important role in the development of EAU and related immune response. More studies are needed to further dissect the biologic activities of this molecule, which may be a promising drug target for T cell-mediated pathogenic immune processes.
